# Postoperative Outcomes Among Dialysis Patients Undergoing Hip Fracture Repair

**DOI:** 10.1177/21514593231195992

**Published:** 2023-08-17

**Authors:** Conover Benjamin M, Wukich Dane K, Sambandam Senthil

**Affiliations:** 1 25989 University of Texas Southwestern Medical School, Dallas, TX, USA; 2 Department of Orthopaedic Surgery, 12334 University of Texas Southwestern Medical Center, Dallas, TX, USA

**Keywords:** hip fracture, dialysis, chronic kidney disease, end-stage renal disease, geriatric

## Abstract

**Background:**

Geriatric hip fractures are strongly correlated with increased morbidity. Even so, postoperative outcomes following surgical repair of hip fractures for patients with end stage renal disease lack extensive investigation. Chronic kidney disease (CKD) poses unique risks for surgical procedures as it has been associated with several complications. Little information is available regarding the outcomes of patients whose renal function decline necessitates dialysis usage. The purpose of this study was to compare post-surgical outcomes based on dialysis usage among CKD patients requiring hip fracture repair.

**Materials and Methods:**

We used the PearlDiver database to identify hip fracture repair patients diagnosed with stages 3, 4, and 5 CKD. We matched the populations according to patient characteristics and comorbidities. We then compared patient complication rates among dialyzed and non-dialyzed CKD patients following hip fracture repair at 30 days, 90 days, and 1 year following the procedure.

**Results:**

Dialyzed patients were more likely to experience myocardial infarction within 30 days (*P* = .02) and 90 days (*P* = .002). Dialyzed patients suffered cardiac arrest at higher rates within the same time intervals (*P* = .02; *P* = .002). Furthermore, dialysis patients developed sepsis (*P* = .005) and pneumonia (*P* = .005) at higher rates within 30 days of operation. Dialysis patients did not have increased risk of blood transfusion within 30 days of the operation (*P* = .07).

**Discussion:**

We found significant increases in myocardial infarction, cardiac arrest, pneumonia, and sepsis risk among dialyzed CKD patients. Blood transfusion risk failed to reach statistical significance. Our findings are consistent with previous research regarding CKD pathophysiology and associated perioperative outcomes.

**Conclusion:**

Given the drastic decline of renal function among patients on dialysis, our findings may be attributable to decreased glomerular filtration rate in CKD as well as dialysis itself. Regardless, multidisciplinary collaboration should be employed when performing hip fracture repair on a patient who is actively undergoing hemodialysis.

## Introduction

Hip fractures resulting from falls among elderly cohorts strongly correlates with increased morbidity.^1^ However, complications arising from surgical repair of hip fractures for patients with certain comorbidities lack extensive investigation even among medical professionals.

It is estimated that chronic kidney disease (CKD) and end-stage renal disease (ESRD) will increase as much as 68% between 2015 and 2030.^2^ Despite the increasing prevalence of ESRD, mortality of this patient population is declining due to advancements in medical care. Many improvements in renal replacement therapy have occurred since hemodialysis was developed more than 75 years ago, and patients with ESRD are living longer. Consequently, the number of patients with ESRD who experience hip fractures will continue to increase. A study from Scotland reported that CKD stages 3-5 was associated with an increased incidence of hip fracture admission and mortality compared to those with normal glomerular filtration rate (GFR).^3^

Perioperative complications associated with CKD have been well documented. ESRD on dialysis was a significant independent risk factor for 30-day adverse events, ICU care, longer admission, and rehabilitation needs in patients undergoing hip and knee replacement.^4^ Dialysis patients undergoing rotator cuff repair or knee arthroscopy had greater odds of experiencing adverse events, readmission within 30 days, and longer operating times.^5^ One study from the University of Iowa concluded that CKD patients experienced a 2-fold increased risk of pulmonary, infectious, cardiovascular, and renal complications after orthopaedic procedures compared to their counterparts with normal renal function.^6^ Deegan et al^7^ reported that CKD patients were at significantly higher risk of developing prosthetic joint infection following total joint arthroplasty. While these studies establish the increased perioperative risk associated with CKD, few have investigated the effects of dialysis while controlling for CKD diagnosis. To accurately predict patient risk, it is prudent to evaluate what complications arise when dialysis treatment is necessitated, rather than relying on the previously established perioperative risks associated with CKD and ESRD diagnosis alone.

One study published in 2019 found that acute myocardial infarction risk was increased among patients on dialysis compared to CKD patients who were non-dialyzed.^8^ These findings are supported by another international study conducted by the Department of Health and Human Services that found that hemodialysis patients not only suffered increased complication frequency and mortality, but they were also found to be at greater risk of bone fractures.^9^ While these studies established significant considerations for dialyzed CKD patients requiring hip fracture fixation, a comprehensive analysis of the risks associated with CKD requiring dialysis treatment is needed.

The purpose of this study is to investigate the impact of CKD in the setting of dialysis usage on perioperative complication risk following hip fracture repair.

## Materials and Methods

### Data Collection and Study Population

To collect data for this retrospective study, we utilized the PearlDiver patient record database, a compilation of insurance providers, including Medicare, Medicaid, private insurance companies, as well as self-pay. Using the mOrtho151 data subset, we analyzed patient records from 2015 to 2021 involving hospital and physician billing documentation. PearlDiver data is Health Insurance Portability and Affordability Act (HIPAA) compliant through de-identification and thus did not require institutional review board assessment.

We identified patients by diagnoses and procedures performed through ICD-10 and CPT (Table 1) codes. We classified the hip fracture repair cohort as those patients who underwent hip fracture fixation or total hip replacement within 30 days of fracture diagnosis. Furthermore, we classified CKD patients as those with a previous diagnosis of stage 3, 4, or 5 renal disease. Stages 3, 4 and 5 CKD correspond to GFR values of 30-59, 17-29, and <15 mL/min, respectively. After defining CKD diagnosis, we crossmatched these groups with the hip fracture group. We then created a cohort of CKD patients who underwent hip fracture repair while concurrently on dialysis treatment while excluding those whose last dialysis treatment proceeded the hip fracture repair by more than 6 months. This group constituted the experimental group. The control group was conisted of patients undergoing hip fracture repair with a previous CKD stage 3, 4, or 5 diagnosis but no history of dialysis usage prior to the surgical procedure.

**Table 1.** ICD-10 and CPT Code Definitions.

Diagnosis/frocedure	ICD-10/CPT coded
Hip fracture	ICD-10-D-S72011A, ICD-10-D-S72012A, ICD-10-D-S72019A, ICD-10-D-S72143A, ICD-10-D-S72141A, ICD-10-D-S72142A, ICD-10-D-S7221XA, ICD-10-D-S7222XA, ICD-10-D-S7223XA
End stage renal disease	ICD-10-D-N184, ICD-10-D-N185, ICD-10-D-N183
Periprosthetic fracture	ICD-10-D-M9701XA, ICD-10-D-M9702XA, ICD-10-D-M979XXA, ICD-10-D-T84040A, ICD-10-D-T84041A
Hip dislocation	ICD-10-D-T84020A, ICD-10-D-T84021A, ICD-10-D-M24351, ICD-10-D-M24352, ICD-10-D-M24359, ICD-10-D-S73004A, ICD-10-D-S73005A, ICD-10-D-S73006A, ICD-10-D-S73014A, ICD-10-D-S73015A, ICD-10-D-S73016A, ICD-10-D-S73024A, ICD-10-D-S73025A, ICD-10-D-S73026A, ICD-10-D-S73034A, ICD-10-D-S73035A, ICD-10-D-S73036A, ICD-10-D-S73044A, ICD-10-D-S73045A, ICD-10-D-S73046A, ICD-10-D-M24451, ICD-10-D-M24452, ICD-10-D-M24459
Sepsis	ICD-10-D-A021, ICD-10-D-A227, ICD-10-D-A267, ICD-10-D-A327, ICD-10-D-A400, ICD-10-D-A401, ICD-10-D-A403, ICD-10-D-A408, ICD-10-D-A409, ICD-10-D-A4101, ICD-10-D-A4102, ICD-10-D-A411, ICD-10-D-A412, ICD-10-D-A413, ICD-10-D-A414, ICD-10-D-A4150, ICD-10-D-A4151, ICD-10-D-A4152, ICD-10-D-A4153, ICD-10-D-A4159, ICD-10-D-A4181, ICD-10-D-A4189, ICD-10-D-A427, ICD-10-D-A5486, ICD-10-D-B377, ICD-10-D-R6520, ICD-10-D-R6521, ICD-10-D-T8144XA
Dialysis	ICD-10-D-Z992
Hip fracture repair	CPT-27244, CPT-27245, CPT-27125, CPT-27130
Dialysis	CPT-90935, CPT-90937, CPT-90945, CPT-90947, CPT-90999

### Patient Characteristics

In evaluating the patient characteristics of the previously discussed cohorts we studied, the following criteria were analyzed: age, gender, Charleston Comorbidity Index (CCI), Elixhauser Comorbidity Index (ECI), obesity, tobacco use, and diabetes diagnosis. The CCI is frequently used as a reliable and verified scoring methodology in predicting long term prognosis and mortality rates.^10^ This index is calculated by a number of comorbidities, including myocardial infarction, peripheral vascular disease, dementia, COPD, etc. Each comorbidity contributes one integer value to the final CCI score, which is ultimately used to estimate 10-year mortality. Similarly, the Elixhauser Comorbidity Index (ECI), is another validated mortality index composed of 30 conditions known to increase length of stay, hospital expenses, and death. It is calculated in a similar manner as the CCI but includes 30 comorbidities in calculating its final score.^11^

One advantage of the Bellwether-PearlDiver database is the ability to crossmatch experimental cohorts according to numerous classifications to control for confounding factors. Utilizing this tool, we sought to clarify the contribution of dialysis on perioperative complication risk while controlling for other associated comorbidities. We matched the “Hip Fracture Dialysis” and “Hip Fracture Without Dialysis” cohorts based on CCI score, ECI score, diabetes diagnosis, tobacco use, and obesity. These populations were then used to analyze perioperative complication rates following hip fracture repair among the control and experimental groups with and without comorbidity matching. As a result, the comorbidity-matched control and experimental groups have similar values for the previously mentioned variables.

### Post-Operative Complications

We analyzed the following perioperative complication rates between the dialyzed and non-dialyzed groups at 30 days, 90 days, and 1 year for the following conditions: dislocation, surgical site infection (SSI), myocardial infarction, cardiac arrest, deep vein thrombosis (DVT), wound disruption, hematoma, pulmonary embolism, blood transfusion, cerebrovascular accident (CVA), dehiscence, mechanical complications, nerve injury, periprosthetic fracture, sepsis, and pneumonia. The incidence rate for each perioperative complication was calculated and compared between the experimental and control groups.

## Results

### Patient Characteristics

In total, 52,082 patients previously diagnosed with CKD underwent surgical repair of hip fracture according to our ICD-10 and CPT code definition. Among these patients, 1686 were reported to have undergone dialysis treatment within the 6 months prior to hip fracture repair and as such they represented the experimental group. The remaining 50,396 patients had no previous history of dialysis treatment and composed the control group in our analysis. Following CKD and dialysis crossmatching and group matching for comorbidities as described previously, 1620 patients remained in both the dialysis group and those without dialysis treatment. Both the matched and unmatched dialyzed and non-dialyzed groups were then analyzed for complication prevalence.

Patient characteristics for age, gender, CCI score, ECI score, obesity, tobacco use, and diabetes are included in Table 2a (Matched) and Table 2b (Unmatched).

**Table 2a.** Hip Fracture Patient Characteristics-Comorbidity Matched.

Patient characteristics	Dialysis (n = 1620)	No dialysis (n = 1620)	*P*
Age (y), mean (SD)	72.60 (7.54)	76.13 (5.61)	<.0001
Female, n	846 (52.22%)	987 (60.93%)	<.0001
CCI, mean (SD)	7.67 (3.38)	7.69 (3.39)	.8665
ECI, mean (SD)	12.99 (3.73)	12.99 (3.73)	1.000
Obesity, n (%)	326 (20.12%)	321 (19.81%)	.8261
Tobacco use, n (%)	572 (35.31%)	516 (31.85%)	.0372
Diabetes, n (%)	925 (57.09%)	886 (54.69%)	.1676

**Table 2b.** Hip Fracture Patient Characteristics-Unmatched.

Patient characteristics	Dialysis (n = 1686)	No dialysis (n = 50396)	*P*
Age (y), mean (SD)	72.44 (7.61)	75.68 (6.06)	<.0001
Female, n	872 (51.72%)	33977 (67.42%)	<.0001
CCI, mean (SD)	7.97 (3.77)	4.53 (3.40)	<.0001
ECI, mean (SD)	13.22 (3.88)	8.52 (4.37)	<.0001
Obesity, n (%)	344 (20.40%)	7001 (13.89%)	<.0001
Tobacco use, n (%)	598 (35.47%)	13975 (27.73%)	<.0001
Diabetes, n (%)	963 (57.12%)	18885 (37.47%)	<.0001

In an effort to de-identify patient data, PearlDiver output presents ‘-1’ when the number of patients in each cohort is below 11. For that reason, statistical analysis of those cohorts in which the population does not exceed this threshold is not possible.

### Complication Rates

#### Complications at 30 Days

Within 30 days post-operation, the investigation without comorbidity controls had sufficient data to perform statistical analysis for myocardial infarction, cardiac arrest, DVT, wound disruption, blood transfusion, CVA, mechanical complications, sepsis, and pneumonia. Among these, myocardial infarction (*P*=<.0001), cardiac arrest (*P*=<.0001), wound disruption (*P*=<.0001), sepsis (*P*=<.0001), and pneumonia (*P*=<.0001) all demonstrated elevated rates among the dialyzed population compared with those without dialysis treatment. The remaining complications failed to reach statistically significant differences. Following comorbidity matching, myocardial infarction (*P* = .02), cardiac arrest (*P* = .02), sepsis (*P* = .005), and pneumonia (*P* = .005) all maintained their statistically significant elevations in perioperative complication development among dialyzed patients compared with their non-dialyzed counterparts. Tables 3 and 4.

**Table 3.** Complication Rates 30 Days, 90 Days, and 1 Year Post Operation; without Comorbidity Matching.

Complication	Dialysis, n (%)	No dialysis, n (%)	Statistics
Dislocation			
30 Days	-1	117 (.23%)	—
90 Days	-1	212 (.42%)	—
1 Year	11 (.65%)	283 (.56%)	OR – 1.16 (.64–2.13) *P* = .63
Surgical site infection			
30 Days	-1	45 (.09%)	—
90 Days	12 (.71%)	181 (.35%)	OR – 1.99 (1.11–3.57) ***P* = .02**
1 Year	25 (1.48%)	289 (.57%)	OR – 2.61 (1.73–3.94) *P* = <.0001*
Myocardial infarction			
30 Days	143 (8.48%)	1981 (3.93%)	OR – 2.26 (1.90–2.70) *P* = <.0001*
90 Days	226 (13.40%)	2932 (5.81%)	OR – 2.51 (2.17–2.90) *P* = <.0001*
1 Year	343 (20.34%)	4630 (9.19%)	OR – 2.52 (2.23–2.85) *P* = <.0001*
Cardiac arrest			
30 Days	32 (1.89%)	285 (.56%)	OR – 3.40 (2.35–4.92) *P* = <.0001*
90 Days	58 (3.44%)	437 (.87%)	OR – 4.07 (3.08–5.38) *P* = <.0001*
1 Year	100 (5.93%)	715 (1.42%)	OR – 4.38 (3.53–5.43) *P* = <.0001*
Deep vein thrombosis			
30 Days	24 (1.42%)	488 (.96%)	OR – 1.48 (.98–2.23) *P* = .06
90 Days	48 (2.85%)	742 (1.47%)	OR – 1.96 (1.46–2.64) *P* = <.0001*
1 Year	67 (3.97%)	1031 (2.05%)	OR – 1.98 (1.54–2.55) *P* = <.0001*
Wound disruption			
30 Days	23 (1.36%)	245 (.49%)	OR – 2.83 (1.84–4.35) *P* = <.0001*
90 Days	39 (2.31%)	446 (.88%)	OR – 2.65 (1.90–3.69) *P* = <.0001*
1 Year	52 (3.08%)	595 (1.18%)	OR – 2.66 (2.00–3.55) *P* = <.0001*
Hematoma			
30 Days	-1	223 (.44%)	—
90 Days	13 (.77%)	304 (.60%)	OR – 1.28 (.73–2.24) *P* = .38
1 Year	30 (1.78%)	448 (.89%)	OR – 2.02 (1.39–2.93) *P* = .0002*
Pulmonary embolism			
30 Days	-1	207 (.41%)	—
90 Days	-1	325 (.64%)	—
1 Year	-1	427 (.85%)	—
Transfusion			
30 Days	153 (9.07%)	4473 (8.88%)	OR – 1.02 (.86–1.21) *P* = .78
90 Days	198 (11.74%)	4811 (9.55%)	OR – 1.26 (1.08–1.47) *P* = .0027
1 Year	248 (14.71%)	5406 (10.72%)	OR – 1.44 (1.25–1.65) *P* = <.0001*
Cerebrovascular accident			
30 Days	0	87 (.17%)	OR – .17 (.01–2.75) *P* = .21
90 Days	0	188 (.37%)	OR – .08 (.005–1.27) *P* = .07
1 Year	0	283 (.56%)	OR – .05 (.003–.84) *P* = .04*
Dehiscence			
30 Days	-1	-1	—
90 Days	-1	66 (.13%)	—
1 Year	-1	92 (.18%)	—
Mechanical comp			
30 Days	0	44 (.09%)	OR – .34 (.02–5.45) *P* = .44
90 Days	0	85 (.67%)	OR – .17 (.01–2.81) *P* = .22
1 Year	0	155 (.31%)	OR – .10 (.006–1.54) *P* = .09
Nerve injury			
30 Days	0	-1	—
90 Days	0	-1	—
1 Year	0	-1	—
Periprosthetic fracture			
30 Days	-1	218 (.43%)	—
90 Days	-1	338 (.67%)	—
1 Year	18 (1.07%)	455 (.90%)	OR – 1.18 (.74–1.90) *P* = .48
Sepsis			
30 Days	71 (4.21%)	761 (1.51%)	OR – 2.87 (2.24–3.68) *P* = <.0001*
90 Days	154 (9.13%)	1390 (2.76%)	OR – 3.54 (2.98–4.22) *P* = <.0001*
1 Year	284 (16.84%)	2513 (4.99%)	OR – 3.86 (3.38–4.41) *P* = <.0001*
Pneumonia			
30 Days	182 (10.79%)	3275 (6.50%)	OR – 1.74 (1.49–2.04) *P* = <.0001*
90 Days	487 (28.89%)	299 (17.73%)	OR – 2.01 (1.76–2.28) *P* = <.0001*
1 Year	7903 (14.17%)	4889 (9.70%)	OR – 2.46 (2.21–2.74) *P* = <.0001*

**Table 4.** Complication Rates 30 Days, 90 Days, and 1 Year Post Operation; Matched for Tobacco Use, Obesity, Diabetes, CCI Score, ECI Score.

Complication	Dialysis, n (%)	No Dialysis, n (%)	Statistics
Dislocation			
30 Days	-1	-1	—
90 Days	-1	-1	—
1 Year	-1	-1	—
Surgical site infection			
30 Days	-1	-1	—
90 Days	-1	-1	—
1 Year	21 (1.3%)	-1	—
Myocardial infarction			
30 Days	134 (8.27%)	99 (6.11%)	OR – 1.39 (1.06–1.81) *P* = .02*
90 Days	212 (13.09%)	155 (9.57%)	OR – 1.42 (1.14–1.77) *P* = .002*
1 Year	324 (20.0%)	250 (15.43%)	OR – 1.37 (1.14–1.64) *P* = .0007*
Cardiac arrest			
30 Days	31 (1.91%)	15 (.93%)	OR – 2.09 (1.12–3.89) *P* = .02*
90 Days	57 (2.72%)	28 (1.67%)	OR – 2.07 (1.31–3.28) *P* = .002*
1 Year	95 (5.86%)	38 (2.35%)	OR – 2.59 (1.77–3.80) *P* = <.0001*
Deep vein thrombosis			
30 Days	24 (1.48%)	20 (1.23%)	OR – 1.20 (.66–2.19) *P* = .54
90 Days	44 (2.72%)	27 (1.67%)	OR – 1.65 (1.02–2.67) *P* = .04*
1 Year	63 (3.89%)	47 (2.90%)	OR – 1.35 (.922–1.99) *P* = .12
Wound disruption			
30 Days	19 (1.17%)	13 (.80%)	OR – 1.47 (.72–2.98) *P* = .29
90 Days	32 (1.98%)	23 (1.42%)	OR – 1.40 (.81–2.40) *P* = .223
1 Year	43 (2.65%)	26 (1.60%)	OR – 1.67 (1.02–2.73) *P* = .04*
Hematoma			
30 Days	-1	-1	—
90 Days	12	-1	—
1 Year	28 (1.73%)	17 (1.05%)	OR – 1.66 (.90–3.04) *P* = .10
Pulmonary embolism			
30 Days	-1	-1	—
90 Days	-1	-1	—
1 Year	-1	-1	—
Transfusion			
30 Days	144 (8.89%)	175 (10.80%)	OR – .81 (.64–1.02) *P* = .07
90 Days	187 (11.54%)	193 (11.91%)	OR – .96 (.78–1.19) *P* = .74
1 Year	234 (14.44%)	230 (14.20%)	OR – 1.02 (.82–1.24) *P* = .84
Cerebrovascular accident			
30 Days	0	-1	—
90 Days	0	-1	—
1 Year	0	-1	—
Dehiscence			
30 Days	-1	0	—
90 Days	-1	-1	—
1 Year	-1	-1	—
Mechanical comp			
30 Days	0	-1	—
90 Days	0	-1	—
1 Year	0	-1	—
Nerve injury			
30 Days	0	0	—
90 Days	0	0	—
1 Year	0	0	—
Periprosthetic fracture			
30 Days	-1	-1	—
90 Days	-1	13 (.80%)	—
1 Year	18 (1.11%)	16 (.99%)	OR – 1.13 (.57–2.22) *P* = .73
Sepsis			
30 Days	67 (4.14%)	38 (2.35%)	OR – 1.80 (1.20–2.69) *P* = .005*
90 Days	147 (9.07%)	76 (4.69%)	OR – 2.03 (1.52–2.69) *P* = <.0001*
1 Year	267 (16.48%)	143 (8.83%)	OR – 2.04 (1.64–2.53) ***P*** = <.0001*
Pneumonia			
30 Days	167(10.86%)	129 (7.96%)	OR – 1.41 (1.12–1.79) *P* = .005*
90 Days	286 (17.65%)	227 (14.01%)	OR – 1.32 (1.08–1.59) *P* = .005*
1 Year	4563 (28.58%)	373 (23.02%)	OR – 1.34 (1.14–1.57) *P* = .0003*

#### Complications at 90 Days

90 days following the hip fracture repair, dialysis patients were found to have statistically significant elevations in surgical site infection (*P* = .02), myocardial infarction (*P*=<.0001), cardiac arrest (*P*=<.0001), DVT, wound disruption ((*P*=<.0001), sepsis (*P*=<.0001), and pneumonia (*P*=<.0001). Myocardial infarction (*P* = .002), cardiac arrest (*P* = .002), DVT (*P* = .04), sepsis (*P*=<.0001), and pneumonia (*P* = .0003) each maintained statistically significant complication risk augmentation following comorbidity matching.

#### Complications at 1 Year

1 year after operation, surgical site infection (*P*=<.0001), myocardial infarction (*P*=<.0001), cardiac arrest (*P*=<.0001), DVT (*P*=<.0001), wound disruption (*P*=<.0001), hematoma (*P* = .0002), transfusion (*P*=<.0001), CVA (*P* = .04), and pneumonia (*P*=<.0001) exhibited elevated perioperative complication rates. Myocardial infarction (*P* = .0007), cardiac arrest (*P*=<.0001), wound disruption (*P* = .04), sepsis (*P*=<.0001), and pneumonia (*P* = .0003) each preserved their elevated rates compared to the non-dialyzed group following comorbidity matching controls.

## Discussion

Given the morbidity and complications associated with dialysis, its usage is reserved for patients with ESRD. As such, dialysis implementation is associated with dramatic deterioration of renal function even compared to other patients with CKD. Once renal function deteriorates to the point of requiring dialysis, we found that numerous perioperative complication risks are increased. Because of this, dialysis usage can be considered a marker of ESRD.^12^ Given the kidneys’ integral role in maintenance of electrolyte homeostasis and blood pressure regulation, renal dysfunction can be reflected in numerous organ systems. We do not know if this increased perioperative complication risk is due to the loss of renal physiologic control or as a direct result of dialysis treatment itself.

Dialysis patients experienced a 178% increase in sepsis occurrence within 30 days of hip fracture fixation compared with their non-dialyzed CKD counterparts. Additionally, surgical site infection (SSI) among dialyzed patients was significantly elevated within 90 days and 1 year. Because of the dramatically decreased renal function in ESRD, dialysis is required in nearly all cases to prevent patient death. The deficiencies observed in our study are consistent with previous research regarding the effect of renal impairment on the immune system. Infectious disease accounts for 16.8% of deaths in ESRD patients.^13^ While neutrophil quantity increases in ESRD, their phagocytosis and intracellular bactericidal capacity dramatically decrease.^14-16^ This deterioration in immunologic function likely contributes to the elevated surgical site infection and sepsis risk observed among ESRD patients.

Our data demonstrated an increased risk of cardiac complications in dialysis patients compared with non-dialyzed CKD patients. This is consistent with a study from Taiwan that reported a 79% increased risk of acute myocardial infarction after orthopaedic surgery in patients requiring dialysis compared to patients not on dialysis.^8^ Among ESRD patients, these complications are particularly devastating. One large study that evaluated data involving 92% of dialysis patients in the United States reported that overall mortality for dialysis patients within 1 year of myocardial infarction was 59% and nearly 90% at 5 years.^17^ Cardiovascular complications account for 51% of deaths in patients suffering from ESRD.^13^ Considering the elevated risk of perioperative cardiac complications, management by a coordinated multidisciplinary team of specialists should be considered in this high-risk cohort of patients.

Several comorbidities have been closely linked to CKD and deterioration to ESRD, especially hypertension and diabetes. These conditions, combined with aberrant physiologic processes precipitated by CKD itself, contribute to a high prevalence of cardiovascular disease among CKD and ESRD patients. As discussed previously, neutrophil function is significantly impaired in advanced renal disease. Consequentially, these dysfunctional leukocytes contribute to a pro-inflammatory environment.^18,19^ Atherosclerotic plaque formation is markedly increased in CKD patients, increasing the risk of cardiovascular and cerebrovascular thrombosis.^20,21^ Renal osteodystrophy is a consequence of abnormal skeletal remodeling that strives to maintain serum calcium and phosphorus homeostasis.^22^ These abnormalities in calcium homeostasis further contribute to cardiovascular plaque calcification.^23^ These calcified plaques help explain our finding of augmented myocardial infarction risk among dialyzed CKD patients compared to controls.

While myocardial infarction poses a devastating prognosis for patients with deteriorating renal function, arrythmia and cardiac arrest account for 83% of cardiovascular deaths among CKD patients.^14^ Our findings demonstrate that in the unmatched cohort, ESRD patients undergoing hip fracture repair have 3.4 times increased likelihood of cardiac arrest compared with CKD patients not requiring dialysis. After matching the patients for comorbidities, dialysis patients maintained a 2-fold increased likelihood of cardiac arrest compared to non-dialysis patients. The unique physiologic conditions and associated comorbidities resulting from CKD contribute to pathologic cardiac remodeling and subsequent cardiac arrest. As a closely associated comorbidity, hypertension can be considered both a cause and a result of CKD.^24^ Chronic hypertension gradually degrades renal microvasculature, limiting kidney function long term and may progress to CKD. Additionally, CKD can contribute to hypertension by impairing renal volemic control. As a result, CKD and hypertension are heavily correlated. Cyclic hypervolemia secondary to dialysis and hypertension contribute to cardiac stress through increased cardiac preload and increased systemic vascular resistance, respectively.^25^ Moreover, previously discussed atherosclerotic plaque formation common among CKD and ESRD patients contributes to systemic vascular rigidity and resistance, further augmenting cardiac afterload. These hemodynamic abnormalities induce hypertrophic alterations among myocytes and fibroblasts within the cardiac structure. Izumaru et al.^26^ found that as GFR values dimmish, left ventricular hypertrophy and cardiac fibrosis increase. Cardiomyocyte hypertrophy, combined with increased collagen secretion and matrix metalloproteinase activation, contributes to cardiac remodeling in response to CKD-induced hemodynamic changes.^25^ This remodeling inhibits cardiac expansive capacity, contributing to diastolic dysfunction. As these deleterious modifications progress, cardiac function is impaired, and risk of cardiac arrest is augmented.

Given the increased risk of cardiac arrest among CKD and ESRD patients, active involvement of medical specialists such as anesthesiologists, cardiologists, nephrologists, and hospitalists can optimize perioperative care. Additionally, timing of surgery in relation to dialysis should be considered to optimize volume and electrolyte status.

While myocardial infarction and cardiac arrest rates were significantly elevated among dialysis patients at 30 days, 90 days, and 1 year, it is unclear to what extent the hip fracture repair itself augments the risk of myocardial infarction within this timeline compared to ESRD patients’ baseline risk of cardiac complications. Nearly 80% of cardiac complications following orthopedic procedures have been found to occur within just 7 days of the operation.^27^ This suggests that the discrepancy between dialyzed and non-dialyzed patients at 3 months and 1 year is unlikely to be significantly elevated due to the hip fracture fixation. We found that within 14 days of hip fracture repair 8.5% of dialyzed patients and 3.2% of non-dialyzed patients experienced myocardial infarction (*P* < .0001) while 1.2% of dialyzed patients and .4% of non-dialyzed patients experienced cardiac arrest within the same time frame (*P* < .0001) (Table 5). While hip fracture and subsequent repair likely contributes to increased cardiac complications compared to baseline ESRD risk, further research is needed to definitively ascertain a quantitative measurement of risk augmentation as a direct result of hip fracture and subsequent surgical intervention.

**Table 5.** Myocardial Infarction and Cardiac Arrest 14 Days Post Operation; Matched and Unmatched for Comorbidities.

Complication	Dialysis	No Dialysis	Statistics
Matched			
Myocardial infarction	99 (8.89%)	97 (5.99%)	OR – 1.53 (1.17–2.00) *P* = .018*
Cardiac arrest	20 (1.23%)	12 (.74%)	OR – 1.68 (.82–3.44) *P* = .16*
Unmatched			
Myocardial infarction	143 (8.48%)	1588 (3.15%)	OR – 2.98 (2.49–3.56) *P* = <.0001*
Cardiac arrest	20 (1.19%)	209 (.41%)	OR – 3.00 (1.89–4.76) ***P* = <.0001**

Our research also found that dialyzed ESRD patients are not at significantly elevated risk of requiring transfusion within 30 days, 90 days, and 1 year of hip fracture repair when controlled for comorbidities (Table 4). Without comorbidity matching, transfusion risk within 30 days and 90 days of surgery was not significantly different between the dialyzed and non-dialyzed populations (Table 3). Extensive research involving dialysis patients has revealed altered hemostatic processes leading to abnormal bleeding patterns, as well as heightened risk of thrombotic events. In ESRD, normal platelet activation and adhesion is altered, leading to dysfunctional clot formation.^21^ Given ESRD patients’ elevated risk for cardiovascular complications resulting from thrombotic disease, many patients are prescribed unfractionated heparin, warfarin, low molecular weight heparin, among other anticoagulant therapies during dialysis treatment.^28^ Furthermore, aspirin prophylaxis has been demonstrated to decrease occlusive vascular complications.^29^ This, combined with platelet dysfunction associated with ESRD have been found to significantly increase risk of major bleeding events in these patients.^21^ These well-studied patterns corroborate our finding that dialysis usage within 1 year of hip fracture repair is associated with necessitating blood transfusion. However, as stated previously, our data indicate that ESRD patients undergoing dialysis treatment are at no elevated risk of requiring blood transfusion within 3 months of hip fracture repair compared to their non-dialyzed counterparts. This suggests that while long-term bleeding risk is elevated in dialysis patients, there is no statistically significant elevated bleeding risk in the acute surgical setting. The half-life of conventional anticoagulation therapies helps explain this phenomenon. The half-lives of warfarin and unfractionated heparin are about 40 h and 1.5 h, respectively.^30^ While these drugs are utilized to prevent thrombotic events during dialysis treatment, they generally are not utilized outside of the dialysis setting. As such, similar bleeding risk between dialyzed and non-dialyzed groups is expected. Although patient risk should be evaluated on an individual basis and consider anticoagulation therapy status, it appears that dialysis treatment itself has a negligible effect on the need for transfusion.

We found that dislocation and periprosthetic fracture occurrence failed to reach levels of statistical significance between the dialyzed and non-dialyzed cohorts. Given the prevalence of renal osteodystrophy among ESRD patients and associated skeletal manifestations discussed previously, this finding is surprising. A possible explanation is that bone homeostasis is negatively impacted on CKD stages 3-5, even when dialysis is not required.

### Limitations

We acknowledge certain limitations of this study. Retrieval of data from registries is retrospective and not prospective in nature. Since this is a database study, the results rely on the accuracy of data recording and coding, and this could introduce potential error into our analysis and reporting bias. For example, we have found that providers rarely document patient death into the database apart from the underlying cause (cardiac arrest, myocardial infarction, cerebrovascular accident, etc.). As a result, while we can detect documented complications that may have led to patient death, we cannot definitively identify those patients who died within the study period.

Additionally, we could not crossmatch populations for GFR values, so we can’t conclude if the augmented perioperative complication risk is attributable to dialysis itself, or if dialysis is a proxy for advanced renal deterioration. Regardless, renal failure requiring dialysis should elicit increased care from healthcare providers in monitoring complication development, especially cardiac function following hip fracture repair.

## Conclusions

Patients undergoing dialysis treatment prior to hip fracture repair experience increased perioperative complications compared with their non-dialyzed CKD counterparts. These complications include increased sepsis, pneumonia, myocardial infarction, and cardiac arrest. Based on this study dialysis patients were not at significantly increased risk of requiring blood transfusion, experiencing dislocation, or periprosthetic fracture following hip fracture repair. When the dialyzed and non-dialyzed groups were compared without controlling for comorbidities, these complication rates increased dramatically, indicating that diabetes, obesity, tobacco use, and other conditions associated with ESRD play a role in increased perioperative morbidity. Each of these complications should be considered when evaluating patients’ risks associated with the operation.
